# Supplementary material for: Effect of antibiotic medicines availability on adherence to standard treatment guidelines among hospitalized adult patients in southern Malawi
Source: PLoS One. 2023 Oct 31;18(10):e0293562. doi: 10.1371/journal.pone.0293562 (PMC10617696; doi:10.1371/journal.pone.0293562)
Supplement: S1 Table — (PDF) [file pone.0293562.s002.pdf]

Supplementary Material 1

S1 Table. Antibiotic treatment protocols for common bacterial infections in Malawi based on 2015 Malawi Standard Treatment Guidelines

**Disease condition**	**Description/sub-type if applicable**	**First line treatment according to MSTG**	**Second-line/ alternative treatment**
Sepsis		Ceftriaxone 2g IV q24h for 10 days	Ciprofloxacilin 400 mg IV every q12h or 500 mg orally bd plus Benzylpenicillin 2MU IV q6h. Switch to oral Ciprofloxacin 500 mg bd plus Amoxycillin 500 mg tds, or oral Co-amoxiclav 625 mg tds, when improved. If intraabdominal source, add Metronidazole 500 mg IV or 400 mg orally tds.
Meningitis	Suspected cases	Benzyl Penicillin 5MU IV or IM stat	
	Confirmed bacterial meningitis	Ceftriaxone 2g IV q12h	Chloromphenical 1g IV q6h or Benzyl penicillin 5MU IV q6h
	Meningococcal Meningitis (Prophylaxis)	Ciprofloxacin 500mg stat.	Give Doxycycline 300mg stat
Pneumonia	Community acquired Pneumonia	Amoxycillin 500mg tds for 5-7 days	Erythromicin 500mg qid for 5-7 days or Doxycycline 100mg bd for 5 - 7 days
	Atypical Pneumonia	Erythromycin 500mg qid for 5 days	
	Nosocomial Pneumonias	Ceftriaxone 2g IV q24h or oral Co-amoxiclav 625mg tds for 7 days	
	Severe Pneumonia	Give Ceftriaxone 2g IV q12h plus Azithromycin or Erythromycin 500mg qid	Give Co-amoxiclav 1.2g IV q8h or Erythromycin 500mg qid for 7 days<br>Or Doxycycline 100mg od for 7 days. If aspirating Add Clindamycin 600mg qid or Metronidazole 400mg tds 1.2g IV q8h
Other respiratory infections	Bacterial Sinusitis	Amoxycillin 500mg tds for 7 days	Erythromycin 500 mg qid for 7 days
	Acute bronchitis	Amoxycillin 500mg tds for 5 days	Doxycycline 200mg on first day

	Bronchiectasis	Co-amoxiclav 625mg tds	Doxycycline 200mg stat then Doxycycline. If sputum is smelling, add metronidazole 400mg tds
	Pharyngitis, Tonsillitis and its complications	Benzathine Penicillin 1.2 MU single dose	Amoxycillin 500mg tds for 7 days or Erythromycin 500mg qid for 7 days
	Peritonsillar Abscess	Amoxycillin 500mg tds and Metronidazole 400mg tds	Benzylpenicillin 2 MU IV q6h. Switch when possible (usually after<br>48-72 hours) to oral Amoxycillin<br>500mg tds or if penicillin allergic Give Erythromycin 500mg qid
	Retropharyngeal Abscess	Give Co-amoxiclav 625mg tds (or Co-Amoxclav 375mg plus Amoxicillin 240mg for 14 days)	Chloramphenicol 25 mg/kg tds, initially IM or IV. later orally for
Urinary tract infections	Cystitis/Urethritis	Ciprofloxacin	Give Nitrofurantoin 100mg qid with food for 7 days
	Complicated Urinary Tract Infections	Give Ciprofloxacin 500mg orally bd for 5 days	Give Co-amoxiclav 375mg tds or 625mg bd for 5 days
Sexually transmitted diseases	Genital ulcer disease (GUD)	Ciprofloxacin 500mg orally stat and Benzathine penicillin 2.4 MU IM stat plus Acyclovir 800mg bd for 7 days	Give Erythromycin 500mg qid for 15 days plus Acyclovir 800mg bd for 7 days
	Early Syphillis in adults	Benzathine Penicillin one dose of 2.4 MU IM	Doxycycline 100mg bd for 15 days or Erythromycin 500mg qid for 15 days
	Late syphilis in adults	Benzathine Penicillin 3 doses of 2.4 MU IM at weekly intervals	Doxycline 100mg orally every 12 hours for 30 days
	Neurosyphilis	Give Benzylpenicillin 4MU IV q6h for 14 days then Give Benzathine Penicillin 2.4 MU IM once weekly for 3 consecutive weeks	Doxycycline 200mg every 12 hours for 30 days
	Trichomoniasis, vaginal	Give Metronidazole 5 mg/kg every 8 hours for 5 days	

	Urethral Discharge/Urethritis	Gentamycin 240mg IM stat plus Doxycycline 100mg bd with food for 7 days	Erythromycin 500mg qid for 7 days
	Abnormal Vaginal Discharge in Women	Gentamycin 240mg IM stat plus Doxycycline 100mg bd with food for 7 days, plus metronidazole 2g orally single dose	
	Lower abdominal pain in women (LAP syndrome)	Gentamycin 240mg IM stat plus Doxycycline 100mg bd with food for 7 days, plus metronidazole 400mg tds for 7 days	
	Acute scrotal swelling or pain	Give Gentamycin 240mg IM stat and Doxcycline 100mg bd for 7 days.	
Skin infections	Cellulitis	Give Flucloxacillin 125 -500mg qid for 7 -10 days	Erythromycin 500mg qid for 7 -10 days
	Staphylococcal Scalding Skin Syndrome	Cloxacillin 250 - 500 qid for 5 days	
Gastrointestinal	Peptic Ulcer Disease/Gastritis	Metronidazole 400mg every 8 hours for 7-10 days ,plus Amoxycillin 1g every twelve hours for 7 - 10days, plus Omeprazole 40mg once daily for 2 weeks	Metronidazole 400mg every eight hours plus, Clarithromycin 500mg twice daily for 7-10 days, plus Omeprazole 40mg once daily for 2 weeks
