# Supplementary material for: Effect of antibiotic medicines availability on adherence to standard treatment guidelines among hospitalized adult patients in southern Malawi
Source: PLoS One. 2023 Oct 31;18(10):e0293562. doi: 10.1371/journal.pone.0293562 (PMC10617696; doi:10.1371/journal.pone.0293562)
Supplement: S2 Table — (PDF) [file pone.0293562.s003.pdf]

Supplementary Material 2

S2 Table. Data collection forms

ASSESSMENT OF AVAILABILITY OF ANTIBIOTIC MEDICINES

Name of District: ____Date of visit: ________/__________/_________

Name of facility: ____Govt ☐ CHAM ☐ Pvt ☐

Main Storage type: ORDINARY ☐ PREFABRICATED SlaB ☐

	**Name of antibiotic Medicine**	**Procurement type**	**Appropriate level of facility (according to the MEML)**	**Stock at Hand**	**NUMBER OF STOCK OUT DAYS (last six month)**	Monthly consumption (in tabs/vials, based on last six months)	Stockout time in last 6 months (= total number of days when this medicine was not available)	**Comments**
						**Take picture for stock card for reference**		
1	Gentamycin	CMST☐<br>PVT ☐	YES☐<br>NO ☐					
2	Ceftriaxone	CMST☐<br>PVT ☐	YES☐<br>NO ☐					
3	Erythromycin	CMST☐<br>PVT ☐	YES☐<br>NO ☐					
4	Azithromycin	CMST☐<br>PVT ☐	YES☐<br>NO ☐					
5	Clarithromycin	CMST☐<br>PVT ☐	YES☐<br>NO ☐					
6	Metronidazole	CMST☐<br>PVT ☐	YES☐<br>NO ☐					
7	Amoxicillin	CMST☐<br>PVT ☐	YES☐<br>NO ☐					

8	Cloxacillin	CMST☐<br>PVT ☐	YES☐<br>NO ☐					
9	Flucloxacillin	CMST☐<br>PVT ☐	YES☐<br>NO ☐					
10	Benzyl penicillin	CMST☐<br>PVT ☐	YES☐<br>NO ☐					
11	Benzathine penicillin	CMST☐<br>PVT ☐	YES☐<br>NO ☐					
12	Ciprofloxacin	CMST☐<br>PVT ☐	YES☐<br>NO ☐					
13	Nalidixic acid	CMST☐<br>PVT ☐	YES☐<br>NO ☐					
14	Cotrimoxazole	CMST☐<br>PVT ☐	YES☐<br>NO ☐					
15	Doxycycline	CMST☐<br>PVT ☐	YES☐<br>NO ☐					
16	Meropenem	CMST☐<br>PVT ☐	YES☐<br>NO ☐					

APPROPRIATENESS OF ANTIBIOTIC THERAPY AND CLINICAL OUTCOMES

1. PATIENT INFORMATION

Patient's study ID:		Age:	Gender: ☐Male ☐Female	Weight (kg):<br>Height (cm):

2. TREATMENT (indicate if there was switching of antibiotics)

Number of antibiotics prescribed: ☐

Antibiotic name (And indication)	Dose, frequency and duration	Recommended dose range	Culture done	Sensitivity test	No. of days taken before antibiotic was administered	Batch Number administered	Batch sampled for test	Comment(s)
			YES☐ NO ☐	+ve☐-ve ☐<br>Not done ☐			YES☐ NO ☐	
			YES☐ NO ☐	+ve☐-ve ☐<br>Not done ☐			YES☐ NO ☐	
			YES☐ NO ☐	+ve☐-ve ☐<br>Not done ☐			YES☐ NO ☐	
			YES☐ NO ☐	+ve☐-ve ☐<br>Not done ☐			YES☐ NO ☐	

CONCOMITANT DRUGS

Drug name	Indication	Dose, frequency and duration	Recommended dose range	Known Interactions with antibiotics prescribed	Comment

3. CLINICAL OUTCOMES

☐ Recovered or recovering ☐ Not recovered or not recovering ☐ Adverse event detected ☐ No adverse event detected ☐ Death

LENGTH OF HOSPITAL STAY (DAYS): ☐

DESCRIPTION OF ADVERSE DRUG EVENTS (INCLUDING TREATMENT FAILURE) IF DETECTED:

RELEVANT MEDICAL HISTORY OF PATIENT: including pre-existing medical conditions (allergies, previous exposure, alcohol use etc.)
