# Supplementary figures and images for: Effect of antibiotic medicines availability on adherence to standard treatment guidelines among hospitalized adult patients in southern Malawi
Source: PLoS One. 2023 Oct 31;18(10):e0293562. doi: 10.1371/journal.pone.0293562 (PMC10617696; doi:10.1371/journal.pone.0293562)

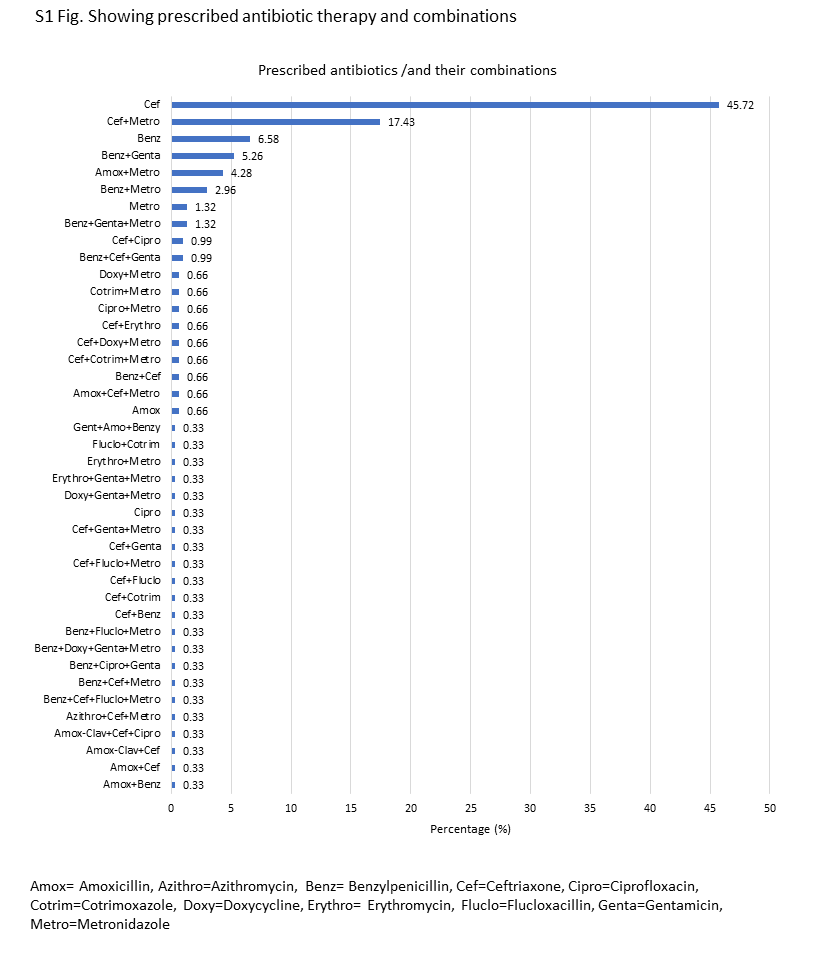

Supplement: S1 Fig — (PNG) [file pone.0293562.s004.png]
