# Supplementary material for: Effect of antibiotic medicines availability on adherence to standard treatment guidelines among hospitalized adult patients in southern Malawi
Source: PLoS One. 2023 Oct 31;18(10):e0293562. doi: 10.1371/journal.pone.0293562 (PMC10617696; doi:10.1371/journal.pone.0293562)
Supplement: Attachment — Submitted filename: Response to reviewer _PLOS_12_10_2023.pdf [file pone.0293562.s007.pdf]

KAMUZU UNIVERSITY
OF HEALTH SCIENCES

Vice Chancellor
Prof. M. Mallewa (BMedSc, MBBS, MRCP, MRCPCH, DTM&H, PhD)
Our Ref:
Your Ref:

13th October 2023

To: The Editor,

Plos One

Dear Sir/Madam,

RE: Response to reviewer's comments

Thanks for your e-mail of 11 October 2023, and for the reviewer comments. We have revised the manuscript, considering all comments by the reviewers. We would like to respond to the reviewer comments as follows:

Section A: Journal requirements:

1. Please review your reference list to ensure that it is complete and correct. If you have cited papers that have been retracted, please include the rationale for doing so in the manuscript text, or remove these references and replace them with relevant current references. Any changes to the reference list should be mentioned in the rebuttal letter that accompanies your revised manuscript. If you need to cite a retracted article, indicate the article's retracted status in the References list and also include a citation and full reference for the retraction notice.

   Response: We have reviewed the reference list and have changed the citation to conform to PLOS guidelines

Section B: Responses to reviewer #1

2. Reviewer #1: See my minor comments / changes/ highlighted in the main text .

Response: We have revised accordingly and replaced the word "Impact" with "effect" in the title as suggested.

We have also made all necessary editing requested throughout the document.

On the issue of benzathine penicillin, we have corrected all to "benzathine benzylpenicillin" as suggested by the reviewer

3. In the Supplementary Material for their revised manuscript, the authors have now included S1 Figure to show the frequency of the use of combination therapies. The figure is fine in principle, but needs correction of a serious technical problem: apparently, half of the names for antibiotics or antibiotic combinations on the left side got lost. E.g. in their revised manuscript, lines 285-286, the authors state: "ceftriaxone and metronidazole (17.4%, n=53); benzyl penicillin and gentamicin (5.3%, n=16)". However, in S1 Figure, while the columns for 17.4% and 5.3% can be found, but they are not labeled with the names of the respective antibiotic combinations.

We have formatted S1 Figure to show the hidden axis labels (S1 Figure revised)

Yours sincerely,

Assoc. Prof. Felix Khuluza

Mahatma Ghandi Road ● P/Bag 360, Chichiri, Blantyre 3 ● Tel: +2651 810 911 / +2651 811 286 ● Email: registrar@kuhes.ac.mw

www.kuhes.ac.mw @KuHeS_mw
